# Genotype distribution of cervical human papillomavirus DNA in women with cervical lesions in Bioko, Equatorial Guinea

**DOI:** 10.1186/1746-1596-4-31

**Published:** 2009-09-09

**Authors:** Benjamín García-Espinosa, Ma Paz Nieto-Bona, Sonsoles Rueda, Luís Fernando Silva-Sánchez, Ma Concepción Piernas-Morales, Patricia Carro-Campos, Luís Cortés-Lambea, Ernesto Moro-Rodríguez

**Affiliations:** 1 Department of Histology and Anatomical Pathology, Rey Juan Carlos University School of Medicine. Av de Atenas s/n. E28922 Alcorcón, Madrid, Spain; 2 Genómica S.A.U., Madrid, Spain; 3 Non-Governmental Organization "Mujer y Madre", Madrid, Spain; 4 Hospital de Móstoles, Madrid, Spain

## Abstract

**Background:**

The HVP vaccine is a useful tool for preventing cervical cancer. The purpose of this study is to determine the most frequent HPV genotypes in Equatorial Guinea in order to develop future vaccination strategies to apply in this country.

**Methods:**

A campaign against cervical cancer was carried out in the area on a total of 1,680 women. 26 of the women, following cytological screening, were treated surgically with a loop electrosurgical excision procedure (LEEP). Cases were studied histologically and were genotyped from paraffin blocks by applying a commercial kit that recognized 35 HPV types.

**Results:**

Cytological diagnoses included 17 HSIL, 1 LSIL, 5 ASC-H and 3 AGUS. Histological diagnosis resulted in 3 cases of microinvasive squamous cell carcinoma stage IA of FIGO, 9 CIN-3, 8 CIN-2, 2 CIN-1, 3 flat condylomas and mild dysplasia of the endocervical epithelium. Fifteen of twenty-five cases genotyped were positive for HPV (60%). HPV 16 and 33 were identified in four cases each, HPV 58 in two other cases, and HPV 18, 31, 52, and 82 in one case, with one HPV 16 and 58 coinfection.

**Conclusion:**

The frequency of HPV types in the African area varies in comparison to other regions, particularly in Europe and USA. Vaccination against the five most common HPV types (16, 33, 58, 18, and 31) should be considered in the geographic region of West Africa and specifically in Equatorial Guinea.

## Background

Cervical cancer is the second most common malignancy in women worldwide, and contributes to 9.8% of all female cancers [1]. In sub-Saharan Africa, cervical cancer is the leading cancer among women [2].

Human papillomaviruses (HPVs) are a group of viruses associated with benign and malignant neoplasms of cutaneous and mucosal epithelia [3]. It is estimated that 6% of the 9 million cancer cases registered each year worldwide could be attributed to HPV infection [4]. The classified high-risk HPVs (HR HPVs) are causally associated with invasive cervical cancer, but they also seem to play a role in the pathogenesis of other invasive cancers, including the anus, vulva, esophagus and conjunctiva [5].

More than 100 HPV types have been identified, and about 40 can infect the genital tract [6]. Currently it is accepted that all squamous cell cervical carcinomas contain at least one of the 18 HPV considered oncogenic high-risk types [7]. Fifteen of them have been ranked as "sure oncogenic risk" types (HPVs 16, 18, 31, 33, 35, 39, 45, 51, 52, 56, 58, 59, 68, 73 and 82), and the other 3 have been classified as "high probable oncogenic risk" types (HPVs 26, 53 and 66) [6].

HPV 16, HPV 18, HPV 31, HPV 58 and HPV 52 are the five most common HPV types in cytologically normal women. They also cause 50% of all HPV infections [1]. However, certain HPV genotypes from cervical cytologic samples vary highly between different geographical regions and show a strong correlation with cervical cancer incidence [8]. HPV 53 is included within the five HPV types most commonly found in eastern Africa [1].

HPV has a strong association with cervical carcinoma and its precursor lesions. In particular, the presence of HPV precedes and predicts the development of a squamous intraepithelial lesion (SIL) [9]. Among low grade squamous intraepithelial lesions (LSILs) where HPV is present, the most common types are HPV 16 (26.3%), HPV 31 (11.5%), HPV 51 (10.6%) and HPV 53 (10.2%). In Africa, however, the probability for an HPV-positive LSIL to be present with HPV type 16 is lower than in Europe [10].

Moreover, the progression of LSIL to a malignant lesion is clearly different depending on the type of HPV infection. Both LSIL HPV 18 positive or HPV 16 positive have a higher probability of progression to cervical cancer than those containing other types of HPV. As a result, it is very important to know the type of HPV infection in order to differentiate women with a high risk of developing cancer from those with lower risk (who could be monitored carefully over a long period of time) [10].

Worldwide, HPV types 16 and/or 18 are detected in more than 50% of all squamous intraepithelial high-grade lesions (HSILs), 70% of infections are detected in invasive cancer and 81.5% of infections are detected in adenocarcinomas [1]. In squamous cell carcinoma (SCC), the most common HPV types are HPV 16 (54.3%), HPV 18 (12.6%), HPV 33 (4.3%), HPV 45 (4.2%), HPV 31 (4.2%), HPV 58 (3%), HPV 52 (2.5%) and HPV 35 (1%) [10]. These types are found in 95% of SCC cases that are positive for HPV DNA [6].

All of the above implies that an effective vaccine against the five most common HPV types (16, 18, 33, 45 and 31) could prevent approximately 90% of cervical cancer cases that occur worldwide. However, regional variations in the distribution of certain types of HPV should be considered in the development of vaccines tailored to different geographic regions [6]. As a result, there is a need to study the prevalence of different HPV types in different geographic areas and, particularly, in less studied regions such as Africa.

## Methods

### Specimen collection and diagnosis

Samples were obtained from a cervical cancer screening campaign carried out on the island of Bioko (Equatorial Guinea) between May and October 2006. The study included a total of 1680 women between 15 and 71 years of age. None of the women had been screened previously for cervical cytological abnormalities or HPV infection.

Study procedures were approved by local authorities. An informed consent was obtained and a face-to-face interview was conducted, soliciting information on the women's demographic characteristics and reproductive history. A general physical and detailed gynecological examination were also carried out. Following a visual inspection of the neck of the uterus, exfoliated cervical cell samples were obtained using the Papanicolaou method. Cytological smears were analysed in Madrid (Spain). Abnormalities were classified with the Bethesda classification system as falling within normal limits or reactive cellular changes (normal), atypical squamous cells of undetermined significance (ASCUS), low-grade SIL (LSIL), high-grade SIL (HSIL), or carcinoma.

In the second phase, we performed surgery on those women cytologically diagnosed with ASCUS, high-grade SIL, and carcinoma. Twenty-six out of thirty women (between 22 and 68 years of age) were able to undergo surgery. The surgical treatment chosen was loop diathermy conization; that is, LEEP (Loop Electrosurgical Excision Procedure). The surgical samples were obtained, processed histologically and analyzed in Spain, showing a high positive correlation between the cytological and pathological final diagnosis.

Samples used for the detection and genotyping of HPV were obtained from paraffin blocks. The lesion area of each paraffin block was selected microscopically and punches of the same size (1 mm × 7 mm). One to three different samples were used to extract DNA. Twenty five out of twenty six cases were HPV genotyped. The remaining case ended after being histopathologically studied.

### Detection and genotyping of HPV

Samples were shipped to Genomica Laboratory. HPV-DNA was isolated with K proteinase.

The Clart^® ^Human Papillomavirus (Genomica, Spain) kit was used for HPV DNA genotyping. HPV testing was carried out by personnel who had no knowledge of the subject's medical history or cytologic diagnosis.

All samples were analysed for the presence of the following HPV types: 6, 11, 16, 18, 26, 31, 33, 35, 39, 40, 42, 43, 44, 45, 51, 52, 53, 54, 56, 58, 59, 61, 62, 66, 68, 70, 71, 72, 73, 81, 82, 83, 84, 85 and 89.

HPV detection was conducted using PGMYO9/PGMY11 general consensus primers designed to amplify a 450 bp HPV L1 gene fragment and two different controls: genomic DNA and PCR to insure a feasible assay.

The detection of the amplified PCR product was performed with a new technological platform using a low density Microarray, anchored in 2 ml tube-AT tube. It allows simultaneous detection of multiple molecular markers in the L1 fragment of 35 different HPV types and in the necessary controls to insure a feasible assay. Finally, for each specific probe the array was printed in triplicate, so each sample was identified in triplicate. The entire process was carried out according to the manufacturer's standard protocol.

PCR products were marked with biotin and, after amplification, they hybridised with the respective probes in specific known AT tube areas. Biotin binds to streptavidin-peroxidase after incubation. The addition of the TMB substrate (3,3',5,5'-tetrametilbinzidine) generates an insoluble product after hybridization.

The results were processed using a software program which makes detection, interpretation and reporting for each sample possible.

### Second round of DNA extraction and PCR

The 10 negative samples for HPV detection were re-evaluated. The initial DNA extraction and new samples for each case were obtained, following the same procedures explained above. For the new paraffin embedded samples a commercially available tissue DNA extraction kit was used (Biotools, Cat. No. 21.136/7).

125 ngm of nucleic acid was used for each 50 μl PCR reaction with Taq DNA Polymerase (Perkin-Elmer Hispania). Each PCR reaction contained primers for the intron E6 of the HPV-16 or Beta-globin, following the the protocol described by Shibata et al. (1988) [11]. Beta-globin was used as a control gene. The primers had the following sequence: E6 HPV-16 H1+5'ATTAGTGAGTATAGACATTA3'; H2 -5'GGCTTTTGACAGTTAATACA3' and B-GLOBIN +5'GGTTGGCCAATCTACTCCCAGG3'; -5'GCTCACTCAGTGTGGCAAAG3'. These primers generated intron E6 and Beta-globin products of 109 and 536 base pairs (bp), respectively. DNA for the HPV-16 was amplified for 35 cycles on a thermal cycler (Perkin-Elmer Cetus Intruments, Techne).

The 50 μl DNA amplification reactions for HPV-16 contained 2.5 μl of Buffer I (100 mM Tris-HCL pH 8.3, 500 mM KCl, 15 mM MgCl2, 0.01% (w/v) gelatin - Perkin Elmer Hispania), 0.15 mM of the dNTP mixture, 15 pmol of each primer, 2.5 units of Taq polymerase, and 1 μl of DNA.

Before adding the enzyme, we did a hot start at 95°C for 10 min., followed by 35 cycles: 45 sec. at 96°C for denaturalization, 45 sec at 55°C for annealing, and 50 sec. at 72°C for extension, with a final extension step at 75°C for 10 min. A second PCR amplification was performed in the same conditions with 1 μl of the previous product as a template. The final product was detected in a 3% agarose gel electrophoresis stained with ethidium bromide. Each PCR reaction and the subsequent hybridization contained negative and positive controls. The last one was a CIN-III, positive for HPV-16 obtained by situ hybridization and PCR amplification of the L1 region.

## Results

Cytologic diagnoses included 17 cases of HSIL, 1 case of LSIL, 5 cases of ASC-H and 3 cases of AGUS (Table 1). Histopathological diagnoses resulted in 3 cases of microinvasive squamous cell carcinomas, 9 cervical intraepithelial neoplasia CIN-3, 8 CIN-2, 2 CIN-1, 4 flat condylomas and mild dysplasia of the endocervical epithelium (Table 2). The pathologic examination of surgical specimens showed a high positive correlation (84.6%) between pre-cytological diagnoses and the final surgical pathology.

**Table 1:** Cases by cytologic diagnosis

**Cytologic diagnosis**	**Number of women**
ASCUS	39
ASC-H	7
LSIL	35
HSIL	19
Squamous Carcinoma	1
AGUS	3
Adenocarcinoma	0
	
**Total pathological smears**	104
**Women needing surgery**	30
**Women not found**	4
**Women undergoing surgery**	26

**Table 2:** Cases by histopathological diagnosis

**Histopathological diagnosis**	**Number of cases**
Condyloma	4
Mild dysplasia of the endocervical epithelium	1
Cervical intraepithelial neoplasia (CIN-1)	2
Cervical intraepithelial neoplasia (CIN-2)	8
Cervical intraepithelial neoplasia (CIN-3)	8
Microinvasive squamous cell carcinoma-FIGO 1A	2
Microinvasive squamous cell carcinoma-FIGO IA1	1
	
Total	26

**Figure 1:**
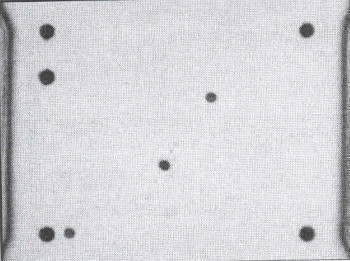
**Low-density array of negative control**. The signals belong to the internal genomic DNA integrity control probes and the plasmid that must be amplified in order to ensure both the correct PCR process and the absence of Taq polymerase inhibitors.

**Figure 2:**
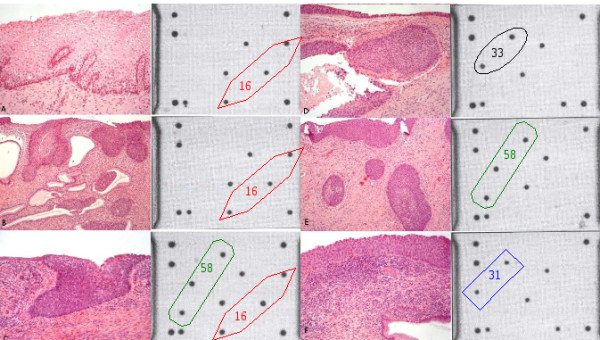
**(A) Case of a 39 year old woman with Condyloma and the low-density array showing an infection with HPV 16**. (B) Case of a 41 year old woman with a **microinvasive carcinoma FIGO IA **showing an infection with **HPV 16**. (C ) Case of a 33 year old woman with a **CIN 3 **and a co-infection with **HPV 16 and 58**. (D) Case of a 68 year old woman with a **CIN 3 **and an infection with **HPV 33**. (E) Case of a 45 year old woman with **microinvasive carcinoma **and the low-density array showing an infection with **HPV 58**. (F) Case of a 39 year old woman with a **CIN 3 **showing an infection of **HPV 31**. Different geometric shapes includes spots corresponding to specific HPV cDNA.

**Figure 3:**
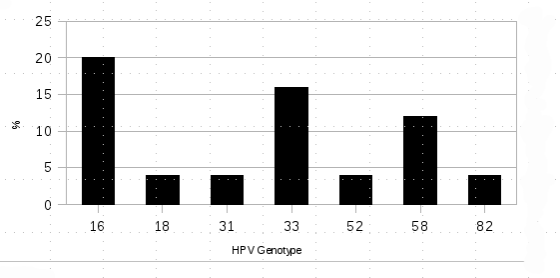
**Human Papillomavirus type distribution in Equatorial Guinea**.

HPV DNA was detected in 60% of samples (Figures 1 and 2). The most frequently detected HPV types were HPV 16 (20% of the positive samples), HPV 33 (16%), HPV 58 (12%), HPV 18 (4%), HPV 31 (4%), HPV 52 (4%) and HPV 82 (4%). We have also found one HPVs 16 and 58 coinfections belonging to a HSIL with CIN-3 (Table 3 and Figure 3). In any case, all these HPV types are considered high-risk oncogenic [6].

**Table 3:** HPV genotyping of samples

**Sample**	**Age**	**Cytology**	**Final Diagnosis**	**HPV detected**							**HPV not detected**	**Beta-globin**
				**16**	**18**	**31**	**33**	**52**	**58**	**82**		
1	41	HSIL	Microinvasive carcinoma	x								
2	45	ASC-H	Microinvasive carcinoma				x					
3	45	HSIL	Microinvasive carcinoma						x			
4	33	HSIL	CIN-3	x					x			
5	68	HSIL	CIN-3				x					
6	47	AGUS	CIN-3	x								
7	39	ASC-H	CIN-3			x						
8	48	HSIL	CIN-3	x								
9	50	HSIL	CIN-3								x	+
10	33	HSIL	CIN-3								x	+
11	48	HSIL	CIN-3								x	+
12	46	HSIL	CIN-2				x					
13	48	HSIL	CIN-2					x				
14	34	ASC-H	CIN-2							x		
15	23	HSIL	CIN-2		x							
16	25	HSIL	CIN-2				x					
17	37	HSIL	CIN-2						x			
18	40	HSIL	CIN-2								x	+
19	30	HSIL	CIN-2								x	+
20	37	ASC-H	CIN-1								x	+
21	30	AGUS	CIN-1								x	+
22	41	AGUS	Mild dysplasia endocervical								x	+
23	39	ASC-H	Condyloma	x								
24	42	HSIL	Condyloma								x	+
25	22	HSIL	Condyloma								x	+
												
			**Total**	5	1	1	4	1	3	1	10	
			**%**	20	4	4	16	4	12	4	40	

In the histological diagnosis we have detected HPV DNA in all microinvasive squamous cell carcinomas. HPV 16, HPV 33 and HPV 58 were found in these samples.

Cervical intraepithelial neoplasia (CIN-1, CIN-2 and CIN-3) had HPV DNA in 63,2% of our cases. HPV 16 and 33 were the predominant types identified in each of the three samples. These rates were followed in frequency by the HPV 58, in two samples. Finally, each HPVs 18, 31, 52 and 82 were only found in one sample.

Twenty-five percent of flat condyloma or mild dysplasia of the epithelium (one out of four samples) showed HPV 16. The cytologic diagnosis of this sample was ASC-H.

We have not found any of the 35 HPV genotypes in a high percentage of samples. Since reactivity for the beta-globin gene excluded DNA absence, we have analysed the most prevalent HPV types and samples corresponding to different cytological and histological diagnosis. Therefore, the results could probably be due to the absence of HPV infection. However, presence of some other HPV type, not detected by our method, or an HPV X should not be rejected. The presence of an uncharacterized type (HPV X) in the African continent has already been suggested in another study in Kenya [2].

## Discussion

In different African regions the prevalence of HPV in women with normal cytology and, therefore, prone to cervical cancer, is very high. In particular, the prevalence of HPV in northern Africa (Morocco) was 21.5%, 31.6% in East Africa (Kenya, Mozambique, Zimbabwe), 17% in West Africa (Nigeria, Senegal) and 15.5% in southern Africa (South Africa) [1].

It has been shown that women with cervical cancer in Africa usually have the following HPV types, in descending order, 16, 18, 33, 45, 35, 31, 58, and 52. On the other hand, HPVs 16, 33, 31, 18, 52, 58, 35 and 56 are those most frequently present in women with HSIL [12].

We have found six of these types in our study (HPVs 16, 33, 58, 18, 31 and 52 in a prevalence order). Our results reinforced the prevalence of 16 and 33 HPV types and highlighted the high percentage of positive cases found for HPV 58 (12%). We also highlight the lack of detection of HPV 45, although its prevalence in cervical cancer has been considered above average in Africa as compared to other geographical areas.

A number of other papers has been published, determining the different HPV types in each African region. Analysis of the data obtained is relevant for the design of effective vaccines against cervical cancer in African regions.

In Kenya (East Africa), a study was carried out on women (with or without pathology) that were clients of a family planning clinic. The study shows that the three most common HPVs types in women with HSIL were: HPVs 16 (37.7% of positive samples), HPV 52 (25.0%) and HPV 35 (17.9%). These researchers also reported an uncharacterized HPV type X in 15.8% of the positive samples [2]. The existence of an HPV X could explain our undetected HPV cases, although infection absence or HPV infection of another undetected type by our method could also be considered.

In another study from Kenya (East Africa), aimed at women with invasive cervical carcinoma (ICC), the most frequently HPV type detected was 16 (43.8% in HIV-negative and 41.2% in HIV-positive women). The second most common type was HPV 18 (nearly 20% in HIV-negative and nearly 30% in HIV-positive). The most frequently found types of HPV in HIV-negative women were the following: HPV 45 (17.0%) and HPVs 35 and 52 (5.2% each). In HIV positive women, the most frequently found types of HPV were: HPV 52 (19.6%) and HPVs 35, 45 and 56 (7.8% each) [13]. This study highlights HPV 45 detection in cases of ICC. Although considered quite prevalent in the African continent, it has not been detected in our work.

HPV 35 was found slightly more often than HPV 16 in Mozambique (southeast Africa), both in women with normal cytology and in those with HSIL or more severe neoplasms [14]. In South Africa, the most frequently found virus in CIN cases was HPV 16 (44%), followed by the HPV types 33 (22%), HPV 18 (19%) and HPV 35 (14%) [15].

In Senegal (northwest Africa), while the rate of LSIL lesions were found to be associated with the detection of HPV types 58 and 16, the risk of HSIL/cancer was found to be associated mainly with the presence of infection with some high-risk HPV types and, in particular, with HPVs 16, 58, 33 and 52, and also with types 31 and 18 [16].

In Ivory Coast (West Africa) women with LSIL, the most frequent HPVs were HPV 16 (12.2%), HPV 18 (6.9%), HPV 31 and 33 (6.1% each) and HPV 70 (5.3%). In women with HSIL from the same region, the most frequent HPVs were HPV 16 (30.6%), HPV 18 (10.2%) and HPVs 33, 58 and 70 (8.2% each) [17]. In Nigeria (West Africa), the most commonly detected types of high-risk HPV were 16 and 35, both in women with no pathology and in women with dysplastic lesions, followed by HPVs 31, 58 and 56 [18].

**Figure 4:**
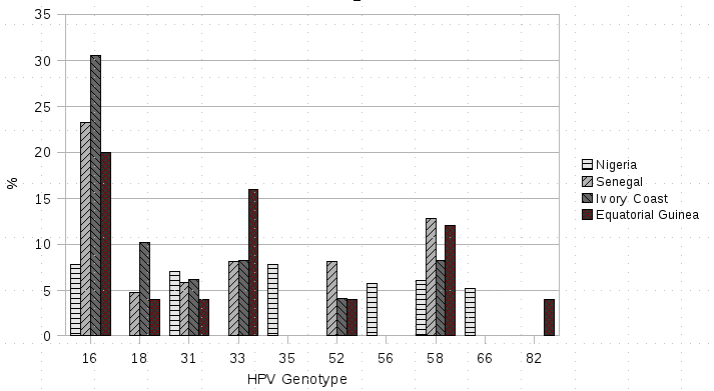
**Human Papillomavirus type distribution in West Africa**.

To sum up, HPV types in women with cervical pathology in Africa are somewhat different from those in Europe and the USA, and they also vary in different regions of Africa. The three most prevalent HPV types were 16, 58 and 33. Other HPVs have appeared quite frequently in our study and have also been persistently detected in previous studies: HPV 18, HPV 31 and HPV 52 (Table 4 and Figure 4).

**Table 4:** High-risk HPVs types more frequent in West Africa

**Country**	**Nigeria^18^**	**Senegal^16^**	**Ivory Coast^17^**	**Equatorial Guinea**
**Pathology**	**Abnormal Cytology/VIA**	**HSIL/cancer**	**HSIL**	**Abnormal cytology**

**HPV types**	16 (7,8%)	16 (23,3%)	16 (30,6%)	16 (20%)
	35 (7,8%)	58 (12,8%)	18 (10,2%)	33 (16%)
	31 (7%)	33 (8,1%)	33 (8,2%)	58 (12%)
	58 (6%)	52 (8,1%)	58 (8,2%)	18 (4%)
	56 (5,7%)	31 (5,8%)	31 (6,1%)	31 (4%)
	66 (5,2%)	18 (4,7%)	52 (4,1%)	52 (4%)

18. Thomas JO et al. Br J Cancer 2004.

16. Xi LF et al. Int L Cancer 2003.

17. La Ruche G et al. Int J Cancer 1998.

The prevalence of HPV 16 in cervical cancer is higher in Europe than in other continents. This may be due to the fact that HPV 16 infection appears to be less favoured by the deterioration of immune status. Therefore, defects in cellular immunity (e.g., parasitic infestation, malnutrition or HIV infection) could contribute to a different frequency of HPV types in certain populations, i.e., those in Africa as compared to other countries [12].

We have concluded that our results are in agreement with studies published on this subject in recent years, and especially with studies in West Africa. We have found the same six most prevalent HPV types in Senegal and Ivory Coast (Table 4). Moreover, prevalence rates are somewhat similar, indicating the reliability of the data and the study method used. Differences found between these studies and the one carried out in Nigeria could be due to the lower severity of lesions. Other areas in which our study overlaps with others include the minor prevalence of HPV 18 and a greater role played by other HPV types such as 58 and 33.

It is estimated that the currently available 16/18 HPVs vaccines could prevent some 350,000 cases of cervical cancer annually. An extension to the following six most common HPV types (31, 33, 35, 45, 52 and 58) could lead to an increase in protection of about 90%, the equivalent of 440,000 cases of preventable cervical cancer per year. It would be a good idea to take regional differences into account when preparing for the inclusion of the HPVs type [12], in case impossibilities arise that prevent the inclusion of the six additional ones in the next generations of HPV vaccines.

## Conclusion

As a result, in the African regions, where the prevalence of different HPV types varies in comparison to what was found elsewhere in the world and particularly in Europe and the USA, the new vaccination strategies against HPV should take into account the most prevalent HPV types in a given population. In fact, other studies have suggested the need to address the HPV 58 during the development of the strategic approach for vaccination against cervical cancer in the geographical region of northwest Africa (Senegal) [16]. Thus, our study reinforces the suggestion that vaccination against HPV 58, and also against HPVs 31 and 33, should be considered in the geographic region of West Africa and specifically in Equatorial Guinea.

### Strengths and weaknesses of the study

Our study gives a valuable description of the most prevalent HPV types in West Africa and confirms other studies found in the same region. The results obtained are relevant due to the scarcity of similar studies conducted in the geographical area in which Equatorial Guinea is located. In fact, the present study is the only one that has been carried out in this country.

The difficulty of obtaining cases is an ostensible weakness due to the number of cases we were, in the end, able to study, as well as the affect this has on the study's significance. It would be of interest to obtain more cases in the future for further study.

## List of abbreviations

HPV: Human papilloma virus; LEEP: Loop electrosurgical excision procedure; ASC-H: Atypical squamous cells-cannot exclude HSIL; AGUS: Atypical glandular cells of undetermined significance; LSIL: Low-grade squamous intraepithelial lesion; HSIL: High-grade squamous intraepithelial lesion; SCC/ICC: Squamous cell carcinoma; invasive cervical carcinoma; CIN: Cervical intraepithelial neoplasia; VIA: Visual inspection of the uterine cervix with acetic acid.

## Competing interests

Genómica S.A.U. (Madrid, Spain) hold the patent of The Clart^® ^Human Papillomavirus kit related to the content of the manuscript.

## Authors' contributions

BGE participated in the design of the study, DNA samples acquisition and analysis, global data analysis and preparation of the first draft. MPNB revised critically the first and final draft, data analysis. SR genotyping data adquisition, carried out the molecular genetic studies. FSS conceived the desing of the study, conceptual and design of the Equitorial Guinea campaign, clinical attendance and surgical performance. MCPM and PCC clinical attendance and surgical performance, data adquisition. LCC planning of the citological studies and data acquisition. EMR conceptual and planning of the design of the study, surgical pathology data acquisition and analysis, final editing. All authors read and approved the final manuscript.
